# Severity Grading and Early Retinopathy Lesion Detection through Hybrid Inception-ResNet Architecture

**DOI:** 10.3390/s21206933

**Published:** 2021-10-19

**Authors:** Sana Yasin, Nasrullah Iqbal, Tariq Ali, Umar Draz, Ali Alqahtani, Muhammad Irfan, Abdul Rehman, Adam Glowacz, Samar Alqhtani, Klaudia Proniewska, Frantisek Brumercik, Lukasz Wzorek

**Affiliations:** 1 Faculty of Computing, University of Okara, Okara 56141, Pakistan; sana.yasin@uo.edu.pk (S.Y.); hafiznasrullah04@gmail.com (N.I.); 2 Department of Computer Science, COMSATS University Islamabad (CUI), Sahiwal Campus, Sahiwal 57000, Pakistan; tariqali@cuisahiwal.edu.pk; 3 Department of Computer Science, University of Sahiwal, Sahiwal 57000, Pakistan; 4 Computer Science Department, CUI, Lahore Campus, Lahore 54000, Pakistan; 5 College of Computer Science and Information Systems, Najran University, Najran 11001, Saudi Arabia; asalqahtany@nu.edu.sa (A.A.); smalqhtani@nu.edu.sa (S.A.); 6 Electrical Engineering Department, College of Engineering, Najran University Saudi Arabia, Najran 61441, Saudi Arabia; miditta@nu.edu.sa; 7 IT Department, Superior University, Lahore 120000, Pakistan; abdulrehman.cs@superior.edu.pk; 8 Department of Automatic Control and Robotics, Faculty of Electrical Engineering, Automatics, Computer Science and Biomedical Engineering, AGH University of Science and Technology, al. A. Mickiewicza 30, 30-059 Krakow, Poland; adglow@agh.edu.pl; 9 Department of Bioinformatics and Telemedicine, Jagiellonian University Medical College, Anny 12, 31-008 Krakow, Poland; klaudia.proniewska@uj.edu.pl; 10 Department of Design and Machine Elements, Faculty of Mechanical Engineering, University of Zilina, Univerzitna 1, 010 26 Zilina, Slovakia; brumercikf@fstroj.uniza.sk; 11 Wzorek. Systems, ul. Kapelanka 10/18, 30-347 Krakow, Poland; lukasz@wzorek.systems

**Keywords:** deep learning, diabetic retinopathy, grading, retinal, fundus images

## Abstract

Diabetic retinopathy (DR) is a diabetes disorder that disturbs human vision. It starts due to the damage in the light-sensitive tissues of blood vessels at the retina. In the beginning, DR may show no symptoms or only slight vision issues, but in the long run, it could be a permanent source of impaired vision, simply known as blindness in the advanced as well as in developing nations. This could be prevented if DR is identified early enough, but it can be challenging as we know the disease frequently shows rare signs until it is too late to deliver an effective cure. In our work, we recommend a framework for severity grading and early DR detection through hybrid deep learning Inception-ResNet architecture with smart data preprocessing. Our proposed method is composed of three steps. Firstly, the retinal images are preprocessed with the help of augmentation and intensity normalization. Secondly, the preprocessed images are given to the hybrid Inception-ResNet architecture to extract the vector image features for the categorization of different stages. Lastly, to identify DR and decide its stage (e.g., mild DR, moderate DR, severe DR, or proliferative DR), a classification step is used. The studies and trials have to reveal suitable outcomes when equated with some other previously deployed approaches. However, there are specific constraints in our study that are also discussed and we suggest methods to enhance further research in this field.

## 1. Introduction

**Figure 1:**
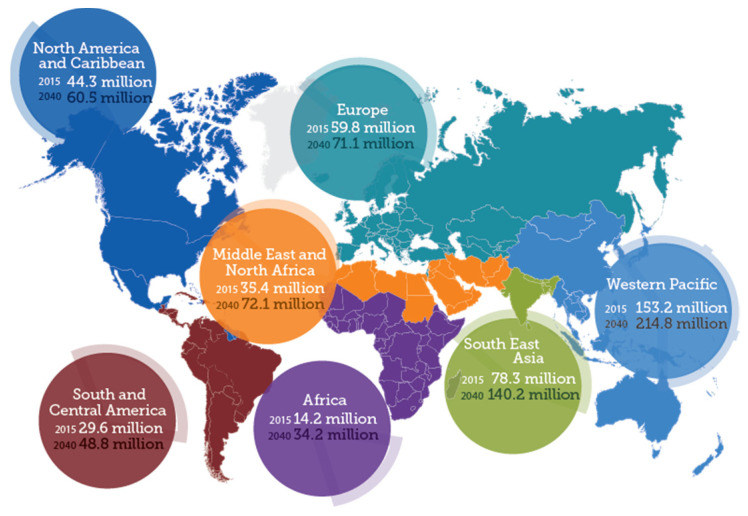
World map of diabetic retinopathy [9].

The data given by the World Health Organization (WHO) show that over 5 to 7 million people across the globe face vision impairment known as diabetic retinopathy (DR), which accounts for around 5–6% of world blindness as described in Figure 1. Timely detection can avert the danger of vision loss.

Automated categorization of cardiovascular and ophthalmologic infections by analysis of fundus images has become a well-known exercise in the field of telemedicine. Previous methods were composed of manual separation; however, it was tiresome, time-consuming, difficult, and skilled manpower is mandatory [1]. On the other hand, computer-aided identification of fundus irregularities is economical, realistic, impartial, and does not need professionally trained ophthalmologists to categorize the fundus images [2]. Improvement in the screening methods is useful in early detection and real-time grading of fundus diseases such as retinitis pigmentosa (RP), diabetic retinopathy (DR), macular bunker, age-related macular degeneration (AMD), retinoblastoma, retinitis pigmentosa, and retinal detachment [3]. A number of template-related, edge-based, and morphological techniques have been used in recent years for autodetection of fundus developments and fundus pathology [4]. Furthermore, numerous unsupervised as well as supervised neural networks (NN)-based approaches have also been used for fundus image examination. Numerous supervised approaches have adopted artificial neural network (ANN), SVM, decision trees (DT), and multilayer perceptron (MLP) [5]. Moreover, filter matching and model-based methods have also been analyzed for the resolution of unsupervised fundus abnormality discovery [4,6,7,8].

At present, identifying diabetic retinopathy (DR) is a time-intensive and manual procedure that involves a qualified and skilled ophthalmologist to examine and assess the colored fundus images of the retina. The human observer most probably can submit his review report a day or two later; therefore, the late review report can lead to delayed follow-up, misinterpretation, or delayed cure. An ophthalmologist can detect DR due to the occurrence of lesions linked with the vascular anomalies initiated by the disease. As this technique is efficient, a number of resources are required. The skill and apparatus needed are often missing in the regions that have a high rate of diabetes in residents, where detection of DR is highly desired. With every passing day, as the cases of diabetes increase, the facilities required to avert vision loss due to diabetic retinopathy will become ever more inadequate.

Due to the lack of physical infrastructure and skilled human resources, the need for an automated and state-of-the-art technique of DR detection has long been advertised, and earlier efforts have shown good improvement using the classification of images, recognition of patterns, and machine learning (ML). By giving digital color fundus images as input, the objective of our work is to establish an automatic computerized system for the detection of DR to every possible limit—preferably making a system with realistic on-ground potential.

**Figure 2:**
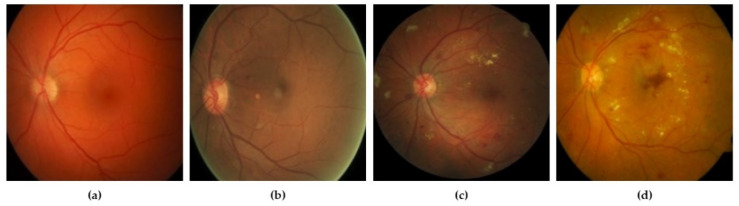
(**a**) Non-proliferative DR mild; (**b**) non-proliferative DR moderate; (**c**) non-proliferative DR severe; (**d**) proliferative DR [14].

DR grades of severity levels are categorized by several lesions identified on the retina, such as microaneurysms, hard exudates, and hemorrhages [10]. By reading the literature, we find various techniques, including deep learning (DL) methods and models that were built for the identification of lesions and classification of DR, exhibiting encouraging results. Khojasth et al. [1] developed a technique of CNN that is used to identify 3 different lesions with the help of 12 layers associated with diabetic retinopathy (DR), i.e., microaneurysms, hard exudates, and hemorrhages. Lam et al. [11] used a method to localize the lesions with the help of various architectures of CNN, i.e., Residual-Net, AlexNet, VGG-16, InceptionV3, and GoogleNet. Eftekhari et al. [4] developed a model of CNN which has 10 layers to identify the microaneurysms lesion. Gersia et al. [6] built a model for the identification of DR that is based on data preprocessing trailed by relating the results of various models of CNN such as VGGNet16 or AlexNet etc. Yung et al. [5] built a model of CNN to categorize levels of DR from normal to severe grade. Pratt et al. [2] built a model of CNN for grading DR with the help of augmentation of data and data preprocessing. Qummar et al. [12] used various preprocessing stages and a combination of different CNN architectures, i.e., ResidualNet50, Inception-V3, Xception, and DenseNet, to categorize DR into appropriate levels. Kori et al. [13] used preprocessing trailed by a model, composed of five ResNets and three different DenseNets, for DR levels. These models have attained high accuracy. Though the developed DL models have shown impressive results in the grading of DR, additional work is required to enhance the precision of grading. Figure 2 represents the various stages of DR mild.

## 2. Related Work

In this related work, we initially debate about various DR techniques for classification. As the dataset which is used in the experimentation is extremely class-imbalanced, and thus has a huge influence on the outcomes of the subjected models, we also discover various techniques in order to discuss this issue. Finally, we discuss the two architectures which directly inspire our model.

As microaneurysms and bleedings are usually the initial indications of diabetic retinopathy, numerous studies have been conducted on these afflictions, mainly for the initial identification of DR. Therefore, we emphasize those methods in our study. Numerous works practice the traditional image processing techniques, and several of them are shortened in Table 1. We have restricted our work in this table to the techniques that identify microaneurysms and bleeding DR, as their occurrence is important for the early recognition of diabetic retinopathy (DR).

### 2.1. Earlier DR Classification Techniques

**Figure 3:**
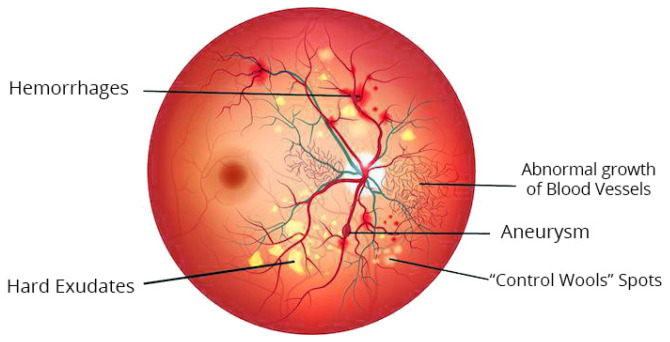
Typical lesions in color fundus image with DR.

Studies on automated DR grading have been a vigorous domain in image processing in the medical field in recent decades [21]. Many scholars have suggested various techniques to categorize diabetic retinopathy (DR). These techniques can be largely categorized into three classes permitting various grading measures: the initial one is the binary, i.e., along with or without diabetic retinopathy; the second one is a 3-stage class of standard, proliferative, or non-proliferative diabetic retinopathy; the recent and most broadly used measure is the 5-class arrangement described and discussed in the previous section. Figure 3 represents the color fundus image with DR.

For binary class, Gardnar et al. [7] used the strength of pixels as feedback features of the model and achieved specificity (Sp) and sensitivity (Se) results of 83.5% and 88.4% correspondingly on a comparatively small dataset of a few hundred photographs. Roychowdhury et al. [22] suggested a 2-step tiered classification method which combines the four machine learning (ML) techniques of SVM, Gaussian mixture models (GMMs), AdaBoost, and k-nearest neighbors (kNNs), and obtained 100% sensitivity results, 0.904 AUC and 53.16% specificity. Prieya et al. classified retinal photographs with diabetic retinopathy into NP-DR or PDR, in which they initially took out blood vessel features and features of hemorrhages as well as exudates. After that, these features were fed into the three grading techniques of SVM, probabilistic neural net (PNN), and lastly the Bayesian classifiers (BC), achieving accuracy of 97.6%, 89.6%, and 94.4%, correspondingly.

For 3-stage classification, Nayek et al. [23] used the properties, i.e., the area of blood vessels and exudates with textures, and fed these features into the neural network (NN). On a very small dataset of 140 photographs, they obtained precision of 93% specificity (Sp) and sensitivity (Se) of 100% and 90% correspondingly. Most of the modern techniques focus on 5-class classification. Achariya et al. [24] used top-order spectrum techniques to obtain features and classified photographs with the help of SVM. This technique achieved accuracy, specificity, and sensitivity of 82%, 88%, and 82%, respectively. Adarish et al. [25] used texture features and area of the infected part of the retina and trained the multiclass support vector machines (SVMs) for classification purposes. With the emergence of deep learning (DL) in previous years, Prat et al. [2] anticipated a model to categorize DR with the help of a 13-layer convolutional net and assessed the results of this network on a large dataset from Kaggle.

### 2.2. Class-Imbalance Feature Learning

In the real-world domain, imbalanced datasets exist, i.e., identifying unreliable telecommunication clients, classification of textual data and retrieval of information from medical imaging, and so on [3]. The majority of algorithms for classification purposes use an objective function that depends upon constant 0–1 and regularized loss function. Without solving the disparity issue, most of the techniques are inclined to be biased for many classes, having poor precision for the smaller classes [8].

Many solutions for the class imbalance problem have been suggested at the data level [3]. However, our proposed solution tries to solve the problem of class imbalance not even on the data level but at the algorithm level as well. For the data level, the proposed solution contains various types of resampling such as undersampling, oversampling, and their combination. At the level of algorithm, a cost-sensitive technique is being used, where every class receives a different weight during the calculation of loss function. In [26], the researcher trained a CNN model with the help of a cost-sensitive loss function to perform saliency identification.

### 2.3. ResNet and Deeply Supervised Nets

In recent years, deep learning (DL) has gradually become widespread in both industrial and academic domains. Different areas such as computer vision recognition of patterns and NLP have observed the considerable power of DNN.

In [27], the researcher presented companion functions in every hidden layer to address the following three problems present in the old-style CNN model: initially, the transparency in intermediate layers for an overall classification; secondly, the robustness and discrimination of learned characteristics, particularly in initial layers; and lastly, the effectiveness of training for vanishing gradient. The idea of a deep space network has been effectively applied to many computer vision problems, i.e., saliency, scene text, and edge identification. In [28], the researchers have shown a ResNet architecture that makes the training easy for very deep neural networks. This model clearly readjusts the layers of architecture as the residual learning function’s place to input, as an alternative to the unreferenced learning function.

## 3. Methodology

**Figure 4:**
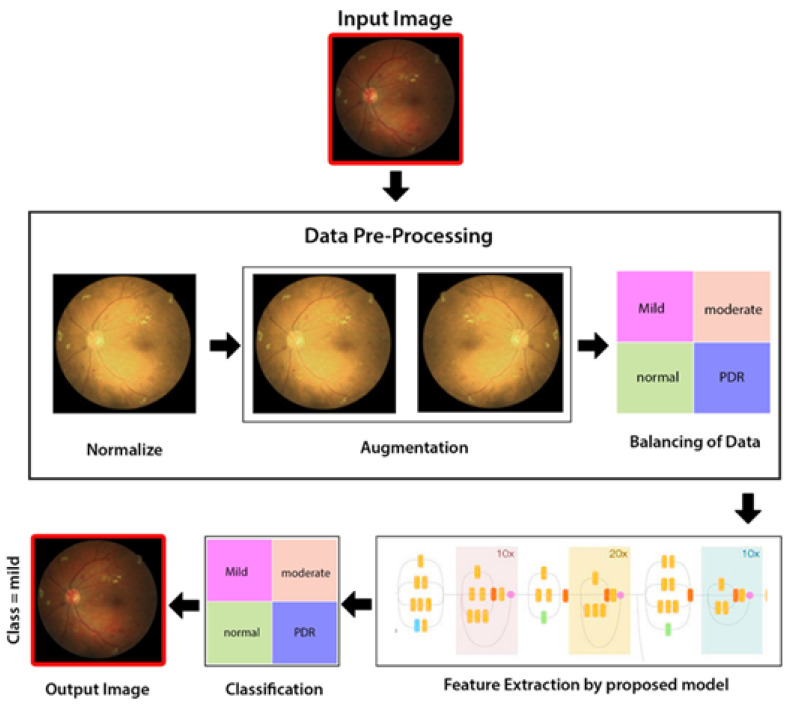
Proposed methodology diagram flow.

As illustrated in Figure 4, the proposed method comprises three stages: a preprocessing level in which intensity normalization is applied, then augmentation of data, and lastly balancing of data and extraction of features through hybrid Inception-ResNet architecture Classification through Neural Network (NN) classifier.

### 3.1. Dataset Collection

Data collection is a significant stage that is often undervalued. The standard of the data given as an input to the system has a robust effect on the resultant performance of the proposed ML model. Thus, it is essential to thoroughly examine the available dataset and consider all potential problems that should be sorted out before going into the modeling phase.

**Figure 5:**
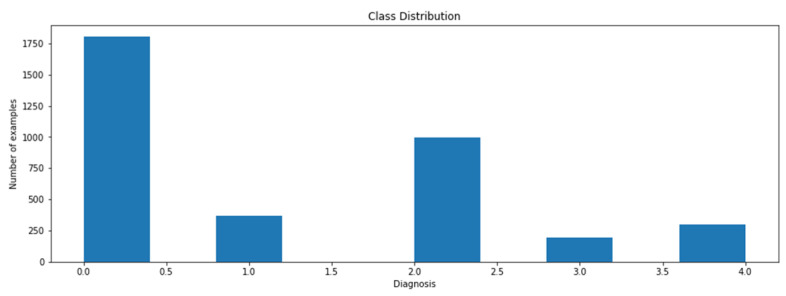
Class distribution.

The data will be collected from multiple online resources such as the IDRiD or Kaggle dataset. IDRiD is the Indian Diabetic Retinopathy (DR) Image Dataset. The dataset comprises 3662 labeled and 1928 test set unknown labeled fundus images of clinical patients, with five different severity level labels, i.e., normal DR, mild DR, moderate DR, severe DR, and proliferative DR (PDR). Figure 5 shows that the data are not balanced: 49% of data are related to the patient with no fundus disease. The other 51% of data represent the various stages of diabetic retinopathy. Class 3 is the least common class (severe), having only five percent of the entire image data.

**Figure 6:**
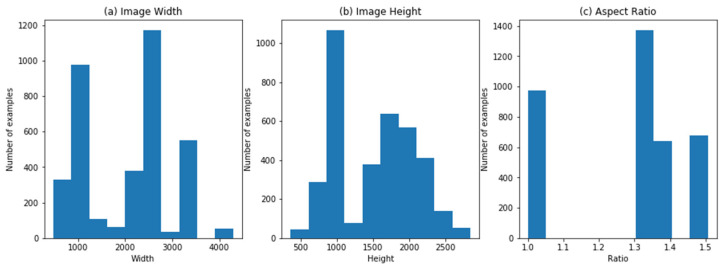
(**a**) Image width; (**b**) image height; (**c**) image aspect ratio.

The dataset is composed of several sources (clinics) with the help of several models of digital cameras, which generate divergences in the resolution of images, width-height aspect ratio, and other constraints which are illustrated in Figure 6, showing the aspect ratio of width and height of images.

### 3.2. Image Preprocessing

To streamline the classification process for the suggested model, it is essential to make sure that all the fundus images look alike.

Initially, due to the usage of various cameras having dissimilar aspect ratios of output images, as a consequence, in some fundus images, there are big black spaces around the fundus. These black regions do not hold any data related to predicting something; hence, they need to be cropped. However, the proportions of black regions differ in every image due to different camera sources. To sort out this problem, we design a special function that transforms the image to grayscale and spots the black regions depending upon the intensity level of pixels. Then, we identify the mask by selecting the rows as well as columns where the pixels surpass the threshold of the intensity level. By doing this, we remove horizontal and vertical boxes that are filled with black color just like the ones detected in the top right of the image. Lastly, after eliminating the black strips, all the images need to be resized to the same width and height.

The second problem is the shape of the eye. On the basis of structure of image, some people have an eye of circular shape, while some people’s eyes seem like an oval shape. As the shape and size of output images after preprocessing positioned in the retina decide the severity level of disease, it is essential to normalize the shape of the eye as well. For this purpose, we design an additional function that helps us to crop an image from the center in a circular shape.

Lastly, with the help of a Gaussian filter, we correct the divergences of brightness and lightening of images by smoothing them.

**Figure 7:**
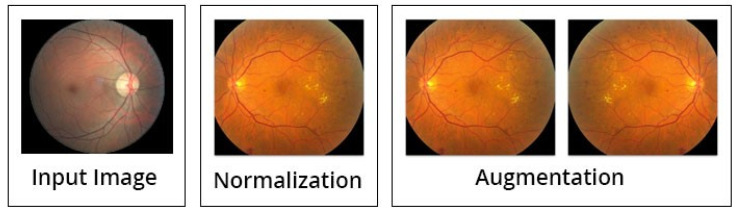
Data preprocessing and augmentation processes [14].

Besides the above mentioned steps, the image preprocessing is executed by three steps; normalization (discussed above in detail), augmentation of images, and lastly the balancing of data (see Figure 7). Initially, the intensities of images are normalized between 0 and 1, by this formula:

$$lm\;{\left(x,y\right)}^{norm}=\frac{lm\;\left(x,y\right)-\min\left(lm\right)}{\max\;\left(lm\right)-\min\left(lm\right)}$$

After the normalization step is completed, the process of augmentation is used to increase the data for training of the model, in order to enhance the standard of the training. This can be achieved by spinning every image around the *y*-axis. The illustration of the suggested preprocessing phases (normalization and augmentation of data) is displayed in Figure 7.

Lastly, with the aim of proficiently training the proposed model, balancing of given data is essential, and this balanced data are provided to the proposed CNN model during the training of the model. In this step, the data are equally split per every stage of DR. This can help in eliminating any unfairness during the process of training the model. Hence, the suggested model trains the network with an equivalent size of fundus photographs, carefully chosen for every grade of DR randomly.

### 3.3. Deep Learning Inception-ResNet Model

The proposed model comprises three phases, which makes this system efficient as compared to other state-of-the-art techniques discussed in this study:

- **Pretraining:** As in medical imaging, the dataset is limited in numbers (N = 3662). That is why by using the transfer learning technique, we train our model twice, firstly on a larger dataset of ImageNet. Although the data of ImageNet are slightly different from the images of retina, the ImageNet dataset might help our network to learn and understand the edges and shapes in the first phase. However, to train the model about target domain of retinopathy in the second phase, we further train the proposed model on a larger dataset featuring around 35,000 retina images.
- **Fine-tuning:** After training the model on ImageNet and the larger retinal dataset, we fine-tune the proposed model on our limited target image set. We make the decisions of modelling depending upon the results of out-of-the-fold estimations.
- **Interpretation:** We sum up the predictions of the proposed model trained on various arrangements of training folds and also use the augmentation of test-time in order to further enhance the performance of the model.

Therefore, the above steps enable us to conclude that firstly we initialize our model with the weights of ImageNet, then train this model on target domain of larger data and lastly fine-tune it on smaller datasets. Results show that our proposed model outperforms the other latest models on smaller datasets.

**Figure 8:**
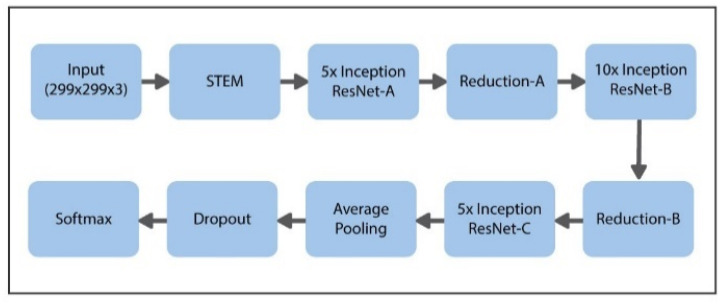
Proposed schema for hybrid Inception-ResNet framework.

The suggested model uses the latest transfer learning (TL) approach with the help of the pretrained Inception-ResNet model, having 50 weighted layers. This pretrained Inception-ResNet model has four phases, every phase is made up of three layers of convolutions, and n times repeated (shown in Figure 6). Inception-ResNet is a CNN structure that is made up of the architecture of the Inception family but includes residual connection (switching the filter concatenation stages of the architecture of the Inception family). This property of the said architecture can help the Inception-ResNet technique to understand the global features specific to data. The comprehensive design of the proposed model is presented in Figure 8.

In order to implement the transfer learning (TL) method, the convolutional layer’s parameters are shifted without any modifications, and the layers that are fully connected are changed with a specially designed classifier that contains four class labels, demonstrating the four diabetic retinopathy (DR) stages. This specially designed classifier is trained with the help of labels obtained from the dataset of IDRiD.

### 3.4. Classification in First Phase

In the first phase, we examined two kinds of specially designed classifiers in order to classify images of DR. The initial classifier is built on the basis of feedforward (NN) pixel-wise classifier, made up of a fully connected (FC) weighted layer among the features vector of Inception-ResNet architecture and the resultant layer that is made up of multiple nodes equivalent to the DR’s stages. The second phase implements a twofold kernel SVMs classification, depending upon the particular feature. In order to train the classifiers of first phase, four labels are commonly used: normal DR, mild DR, moderate DR, and severe/proliferative DR. Severe and proliferative DR have been categorized into the same label, “severe/proliferative DR (PDR)” throughout experimentation, because of the similarity of their fundus images with each other. In order to choose the best classifier, the suggested model has been evaluated with the help of standard metrics for evaluation, i.e., the precision of classification.

### 3.5. Classification in Second Phase

Because of the similarities of the last two stages (severe/proliferative DR (PDR)), the original Inception-ResNet is not capable of differentiating between these two classes. In order to achieve the best accuracy, after the first phase classification, another second phase Inception-ResNet classification is included for this purpose. This phase is trained offline in order to differentiate between these two (severe and PDR) classes, whereas the modified severe and PDR fundus images of the first phase are fed into it. The resultant of this phase is either “severe or proliferative DR”.

### 3.6. Performance Evaluation Metrics

In order to estimate the different metrics of performance for the suggested model, different performance evaluation metrics for the classification’s accuracy (CA) are used. CAg denotes the portion of the properly categorized fundus photographs for the particular grade “g”, described as:

(1) $$\mathrm{CAg}=\frac{correctly\;classified\;grade\;g\;images}{total\;number\;of\;grade\;g\;images}$$

where *g* represents the grades of the diabetic retinopathy (DR), i.e., *g* ∈ (“normal to severe DR”). For example, if 80 from a total 100 normal photographs are categorized properly, then CA_normal_ = 80%. The accuracy of CA of the whole system classification could be obtained as the individual’s average of CAs for every stage.

## 4. Experimental Work

The comprehensive setup of the suggested model, associated outcomes, and results are discussed below.

### 4.1. Dataset and Experimental Setup

For the training of CNN’s architecture of Inception-ResNet, the data are divided in a random manner into 30% testing and 70% training dataset. The process of training reduces the losses of cross-entropy with the help of a learning rate (LR) of 4–10 and 20 epochs at maximum.

### 4.2. First Phase Results

In order to examine the usefulness of every preprocessing step, i.e., normalization of data in the first place then augmentation of data and lastly the balancing of data in the suggested network, results of the network are matched for the initial classification phase, among the six categories:

- **Category A**: There is no preprocessing step. Only uses the unprocessed images.
- **Category B**: Simply the process of normalization is being used.
- **Category C**: Normalization as well as augmentation.
- **Category D**: Normalization as well as balancing of data.
- **Category E**: Implementing the normalization of the dataset, augmentation as well as balancing of data.
- **Category F**: Implementing the full normalization, augmentation as well as balancing. The last two categories include a three-step process.

**Figure 9:**
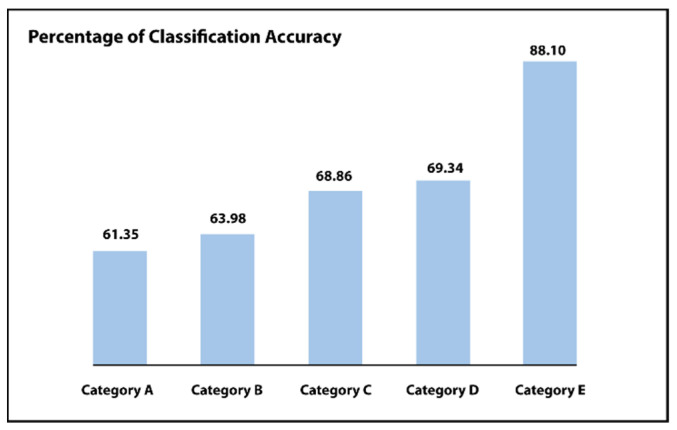
First phase results of Inception-ResNet comparing different categories.

For every category, outcomes are compared among the pixel-wise neural network classifier or a classifier of support vector machine (SVM). Such as shown in Figure 9 and Table 1 and Table 2, the pixel-level neural network classifier delivers improved precision as compared to the support vector machine (SVM) classifier for each examined category (i.e., category A to F). The output of this system progressively improves by growing the utilized preprocessing stages, i.e., with the help of the suggested step of normalization of intensity, which increases the overall classification accuracy from 61.35% to 63.98%. Including balancing or augmentation, the step increases the performance to CA = 68.86% or CA = 69.34%, correspondingly. Lastly, including all the suggested stages increases the accuracy of the suggested system to CA = 84.62% or CA = 88.10%. These outcomes demonstrate the effect of the suggested preprocessing stages for enhancing performance.

### 4.3. Second Phase Results

A second phase Inception-ResNet model is included to differentiate between the two classes (severe/PDR), whereas the adjusted severe or proliferative DR fundus photographs of the initial phase are fed into it. In order to show the benefit of the suggested 2-step model for diabetic retinopathy classification, it is associated with further relevant studies on the similar IDRiD dataset. As illustrated in Table 2 and Figure 9, the performance of the system steadily improves by increasing the utilized steps of preprocessing. For example, with the help of the suggested step of intensity normalization, the classification accuracy increases from 61.35% to 63.98%. Including a balancing step or an augmentation process improves the classification accuracy from 63.98% to 68.86% or classification accuracy = 69.34%, correspondingly. Lastly, including all the anticipated steps increases the accuracy of the proposed model from classification accuracy = 69.34% to classification = 88.10%; these outcomes express the effect of the anticipated steps of preprocessing in order to enhance the performance. As shown in Table 2, the proposed 2-step Inception-ResNet model has shown better performance by implementing all the suggested preprocessing stages.

### 4.4. Analysis of the Performance and Complexity

The benefit of the suggested 2-step Inception-Residual-Net model is its capability to differentiate among all the categories with great performance, which is supported by the value of CA. Furthermore, as we train the proposed model offline, so the overhead to include the second phase Inception-Residual-Net is minimized, taking into consideration that the second Inception-ResNet phase is used only when the result of the initial stage is in the form of class “severe or PDR”. We compare the results of our model with the current state-of-the-art work [10,29,30,31,32] because this model follows the technique that has some resemblance with our proposed model. As in our proposed model, we try different combinations of preprocessing steps and come up with better results after adding the new step every time; this model also implements the preprocessing steps by bringing together eight models of CNN. These above mentioned models are composed of five different-sized ResNets (ResNet18, ResNet34, ResNet50, ResNet101, and ResNet152) and three Inception Nets. As we can see, the above method uses both ResNet and Inception Net. So, it is wise to compare our model with the above model because our model also applies both Inception and ResNet models with fewer layers as compared to the largest ResNet152 of the above technique, which clearly shows that the training process of our proposed Inception-ResNet model has high performance and lower cost of computation. Furthermore, the testing time will be considered short. With the help of a 3.4 Intel CORE i3 and 8 GB of RAM and Python as the programming language, the suggested two-step Inception-ResNet model processes a testing fundus image and delivers its stage, usually an average of 1.32 s that includes 0.21 s for normalization of data and 0.41 s to calculate the first-phase DR stage and 0.41 to give the results of the second-phase DR stage. Table 3 shows the comparison of results between associated techniques.

We are concerned with the problem in which our final output results are in the form of multiple classifications, so for loss function the use of cross-entropy is most suitable. Cohen’s kappa is widely used for evaluation purposes. This evaluation matrix is usually used for the measurement of the agreement between predicted and actual labels. As this evaluation matrix is non-distinct, we cannot use the Kappa as a loss function. Simultaneously, we can use this result to measure the performance of the suggested system. We use an optimizer named “Adam” initially by using a learning rate (LR) of 0.002. Throughout the process of training, we use an LR scheduler that multiplies the LR with 0.5 after each five epochs’ group. This assists in making slighter variations to the weights of the network as we are approaching optimization.

**Figure 10:**
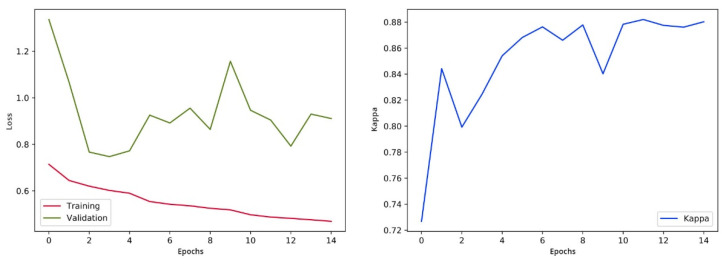
Validation loss and training visualization pre-fine-tuning.

After every epoch during training, we authenticate the proposed model on the selected images. We take out the class score from the last fully connected (FC) layer and forecast the correspondence of image class to the maximum score. The training process is designed for 15 epochs, following the evaluation matrices such as Cohen’s kappa and validation loss. The training process will be stopped if the kappa is unchanged for five successive epochs and weights of the model will be saved for the epoch related to the uppermost validation kappa. The visualization of loss and training is shown in Figure 10.

**Figure 11:**
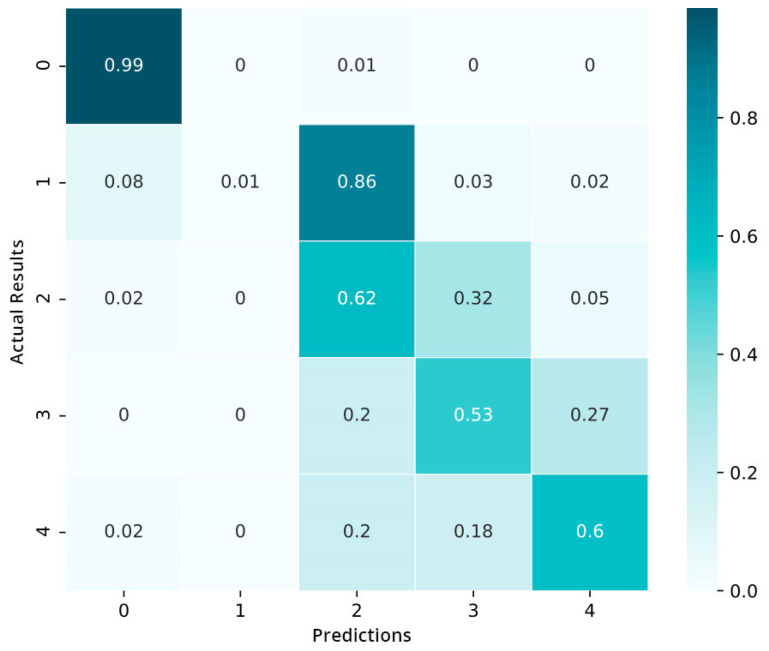
Confusion matrix pre-fine-tuning.

We also build a confusion matrix (CM) of the model that is trained in Figure 11. The values in the cells represent the percentages (%). The preliminary results show that the model is not well capable of distinguishing between the moderate and mild stages of diabetic retinopathy (DR): 85% of data in the mild category are categorized in the moderate category. Only the data of the normal category show an encouraging performance. As a whole, we conclude that the model is inclined to complicate the nearby severity classes but hardly misclassifies the proliferative DR as well as the mild category.

**Figure 12:**
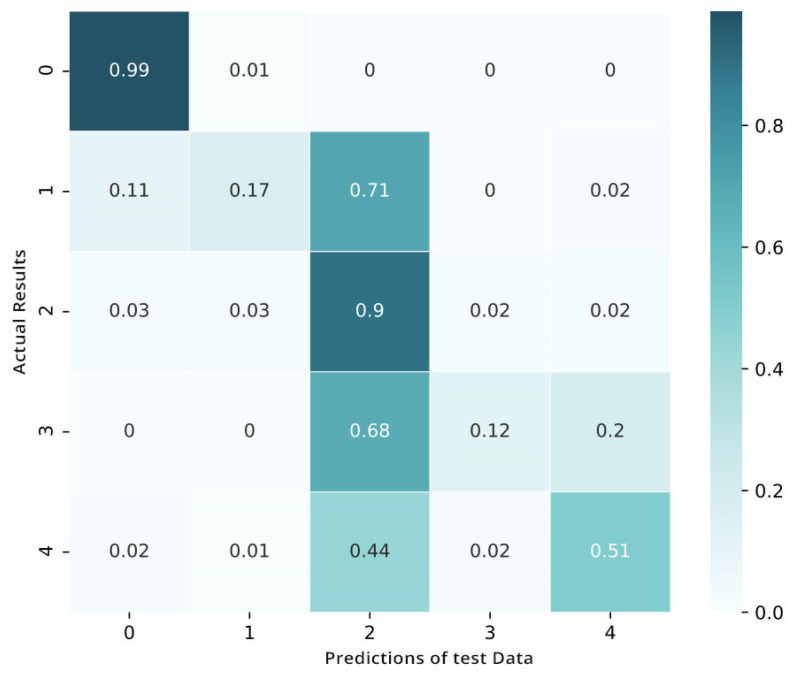
Confusion matrix after fine-tuning.

**Figure 13:**
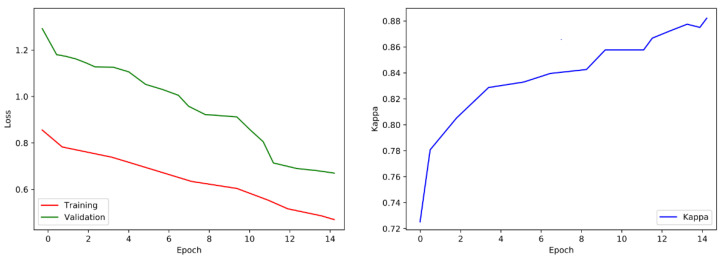
Validation loss and training visualization after fine-tuning.

The process of fine-tuning is performed on the targeted data in four-fold validation. To make sure that we have sufficient samples of every category or stage, we perform cross-validation through the process of stratification. After the fine-tuning, the matrix shows the benefits of the tuned network over the pretrained convolutional neural network and shows an improved performance in categorizing the mild classes of diabetic retinopathy (Figure 12). Yet, when the visualization of loss and training is shown in Figure 13 we also notice that this fine-tuned model categorizes numerous examples as class 2 (moderate).

After training the model we will now offer some estimates regarding the test data. We combined these predictions with the help of the model trained through the loop of cross-validation. For this purpose, from the last FC layer, we take out class scores and express predictions of every class with the highest score. Then, we make an average of the predictions of the four models trained on alternative arrangements of the training folds. Table 4, Table 5 and Table 6 given below show the various arrangements of predictions.

## 5. Conclusions and Future Work

This study presents a three-phase framework to automate the detection grading of DR. The suggested method is composed of preprocessing, extraction of feature, and lastly, classification. Experimental outcomes express that with the help of augmentation and balancing of data we can considerably enhance the performance of the system. Comparing the outcomes with other state-of-the-art work on the IDRiD dataset endorses the greater accuracy of the suggested system for grading of DR. In the future, there will be some ways to improve this method. Initially, the help of bigger architecture and enhancing the number of epochs during the training phase will have an extraordinary probability for improved outcomes. Simultaneously, this would need additional processing power and resources, which seems to be impractical when we use an automated system in real practice. Secondly, the preprocessing methods of target image data could be further enhanced. Lastly, the state-of-the-art results of other researchers are dependent on the assembling of CNN architectures by variations in sizes and architectures. Combining several dissimilar networks and joining together their expected results could also enhance the suggested solution. Our idea is to examine the fundus images with the help of other deep learning techniques to enhance the performance and we will also use other databases and datasets to verify the strength of the suggested system.

## Figures and Tables

**Table 1:** Studies that implement deep learning techniques for the detection of DR.

Title 1	Approach	Databases	Results
Orlando et al., 2018 [15]	Patch-based convolutional NN and conventional candidate selection trailed by random forest (RF) and including the manually made features	Messidor, E-Ophtha, and DIARETDB1	DR Screening on Messidor
			Areas beneath the ROC curve = 0.893 Sensitivity = 0.911
			Need for referral on Messidor
			Area under the receiver’s operating characteristic curve = 0.934 Sensitivity = 0.974
			Areas beneath the ROC curve = 0.893 Sensitivity = 0.954
Chudzik et al., 2018 [16]	CNN based on Patch technique and loss function (LF)	ROC, E-Ophtha, and DIARETDB1	Free-response receiver operating characteristic Curves
Quellec et al., 2017 [17]	Pixel-based visualization with image-based CNN, and classifier named as spatiotemporal features map (STFM)	Private, DIARETDB1, and Kaggle	Required for recommendation on Kaggle test-dataset
			Areas beneath the ROC curve = 0.893 Sensitivity = 0.954
Ting et al., 2017 [18]	Group of eight image-based convolutional NNs which use a variation of the VGG-Net	Personally collected dataset	Screening of diabetic retinopathy
			Area under the receiver’s operating characteristic curve = 0.936
			Required for recommendation
			Areas beneath the ROC curve = 0.893 Sensitivity = 0.958
Gargeya et al., 2017 [19]	Minor image-based convolutional NN, augmentation of data by rotation, contrast, and intensity enhancement, evaluated on more than 75,000 images	EyePACS-1 dataset for training and Messidor-2 dataset for testing	Required for a recommendation of diabetic retinopathy on Messidor-2
			Area under the receiver’s operating characteristic curve = 0.94
			Sensitivity = 0.93
			Specificity = 0.87
Gulshan et al., 2016 [20]	A collection of Inception-V3 architecture that is trained on more than 100,000 images, including many grades for each image and two images for each subject	Messidor-2, Private and (EyePACS)-1	Required for a recommendation of diabetic retinopathy on Messidor-2
			Area under the receiver’s operating characteristic curve = 0.999

**Table 2:** Results comparison for the severity grading of the first phase of four levels of diabetic retinopathy with the help of alternate arrangements.

Category	Preprocessing			Labels	Number of Images	Classification Accuracy	
	Normal	Augmentation	Balancing			Inception-ResNet	State-of-the-Art NN
A	Not used	Not used	Not used	Mild DR	27	0	0
				Moderate DR	171	57.82	71.03
				Normal DR	169	79.00	56.55
				PDR	153	55.52	52.88
				**Final**	**520**	**61.35**	**45.13**
B	Used	Not used	Not used	Mild DR	27	0	0
				Moderate DR	171	28.41	29.51
				Normal DR	169	81.00	78.51
				PDR	153	88.13	81.41
				**Final**	**520**	**63.98**	**46.17**
C	Used	Used	Not used	Mild DR	52	0	0
				Moderate DR	332	36.62	13.77
				Normal DR	334	88.00	97.58
				PDR	310	93.62	83.49
				**Final**	**1028**	**68.86**	**48.46**
D	Used	Not used	Used	Mild DR	30	41.86	71.59
				Moderate DR	25	41.86	12.76
				Normal DR	45	72.43	11.66
				PDR	50	94.43	98.86
				**Final**	**150**	**69.34**	**47.04**
E	Used	Used	Used	Mild DR	55	92.34	92.34
				Moderate DR	55	67.66	21.23
				Normal DR	55	98.95	55.33
				PDR	100	90.95	93.33
				**Final**	**265**	**88.10**	**65.00**

**Table 3:** Results comparison between associated techniques ([10], 2018) for 5-stage DR classification and the suggested 2-phase model with the help of Inception-ResNet relating neural network classifiers.

Techniques	Labels	Data	CA%	
			ResNet	Inception-ResNet
Two-stage proposed system (Case E)	Mild	50	93.33	93.33
	Moderate	50	20	66.67
	Normal	50	53.33	100
	Severe	50	86.66	93.33
	PDR	50	86.67	87.33
	Total	1250	65.00	86.67
Inception/ResNet ensemble	Total Images	56	83.90	

**Table 4:** Predictions.

	0	1	2	3
0	2.0	1.5	2.0	1.5
1	2.5	2.0	2.0	2.0
2	2.0	2.0	2.0	2.5
3	3.0	2.0	2.0	2.5
4	2.0	1.5	2.0	2.0

**Table 5:** Correlational matrix.

	0	1	2	3
0	1.0000	0.9634	0.9563	0.9544
1	0.9634	1.0000	0.9696	0.9480
2	0.9563	0.9696	1.0000	0.9488
3	0.9544	0.9480	0.9468	1.0000

The mean correlation is 0.9673.

**Table 6:** Summary statistics.

	0	1	2	3
Count	1927.00	1927.00	1927.00	1927.00
Std	0.9737	0.9740	0.9706	0.9610
Mean	1.6877	1.7113	1.7162	1.6511
min	0.0000	0.0000	0.0000	0.0000
25%	1.4000	2.0001	2.0001	1.3751
50%	2.0001	2.0001	2.0001	2.0001
75%	2.0001	2.0001	2.0001	2.0001
max	4.100	4.100	4.100	4.100
